# Supplementary material for: Conditional chemoconnectomics (cCCTomics) as a strategy for efficient and conditional targeting of chemical transmission
Source: eLife. 2024 Apr 30;12:RP91927. doi: 10.7554/eLife.91927 (PMC11060718; doi:10.7554/eLife.91927)
Supplement: Figure 3—source data 3. [file elife-91927-fig3-data3.zip › Figure 3 source data3 part 1.zip!/Figure 3-Source data 3-part1/1067-9-CRISPResso_Report_c33kf3u3.zip!/CRISPResso_on_1067_11/9.Alleles_frequency_table_around_sgRNA_agccaggtgtcgcagacgat.pdf]

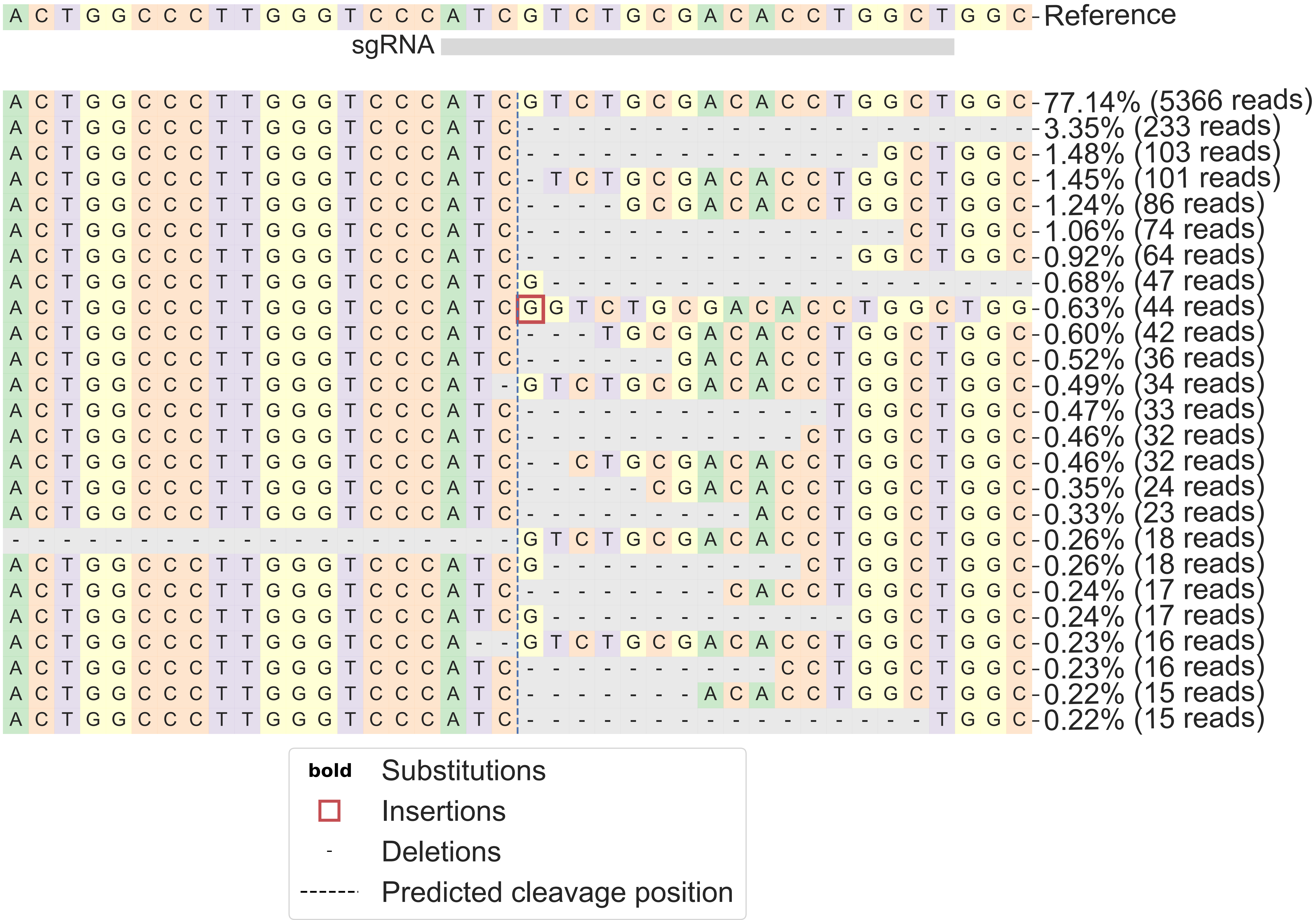
Reference
sgRNA
77.14% (5366 reads)
3.35% (233 reads)
1.48% (103 reads)
1.45% (101 reads)
1.24% (86 reads)
1.06% (74 reads)
0.92% (64 reads)
0.68% (47 reads)
0.63% (44 reads)
0.60% (42 reads)
0.52% (36 reads)
0.49% (34 reads)
0.47% (33 reads)
0.46% (32 reads)
0.46% (32 reads)
0.35% (24 reads)
0.33% (23 reads)
0.26% (18 reads)
0.26% (18 reads)
0.24% (17 reads)
0.24% (17 reads)
0.23% (16 reads)
0.23% (16 reads)
0.22% (15 reads)
0.22% (15 reads)
bold Substitutions
Insertions
Deletions
Predicted cleavage position
